# Effective Light Absorption Using the Double-sided Pyramid Gratings for Thin-Film Silicon Solar Cell

**DOI:** 10.1186/s11671-018-2607-1

**Published:** 2018-07-04

**Authors:** Duan Zhiqiang, Li Meicheng, Trevor Mwenva Chonto

**Affiliations:** 1 School of Mathematical and Physical Science, North China Electric Power University, Beijing, 102206 People’s Republic of China; 2 State Key Laboratory of Alternate Electrical Power System with Renewable Energy Sources, School of Renewable Energy, North China Electric Power University, Beijing, 102206 People’s Republic of China

**Keywords:** Geometric Optical Design, Nanomaterials, Thin Films, Solar Energy

## Abstract

The design of double-sided pyramid grating structure can be used to enhance broadband light absorption. The front grating can greatly reduce the light reflection, especially in the short-wavelength region, and the rear grating can also achieve that same effect in the longer wavelength region. In the paper, for the double-sided pyramid grating structure, the photon absorption distribution of each part is studied and compared with the bare crystalline silicon. Theoretical results show that, by reasonably adjusting the structure parameters of the double-sided grating, the light reflection of the whole band can be reduced greatly which is beneficial for black silicon formation and the total light absorption is also increased. However, further studies have shown that using the rear grating does not improve the effective light absorption of the crystalline silicon.

## Background

With the progress in micro-fabrication technology, nanometer surface morphology and structure design have become more common and really important [1, 2]. The optimization design of parameters has become more urgent and necessary, especially for the crystalline silicon (CS) thin-film solar cells [3–6]. There are some reports on the double-sided grating design applied to CS thin-film solar cells, and all of them have expressed similar opinions that such a structure can achieve broadband light absorption enhancement which is able to reach the Yablonovitch limit [7–10]. There is no doubt that the double-sided grating design can improve the overall light trapping capability of CS solar cells. After all, the generation and separation of electron-hole pairs occurs inside the CS, and considering each absorbed photon with energy greater than the band gap produces one and only one electron-hole pair, so how the photon absorption is distributed among the various parts of the CS solar cell is the focus of this article. In addition, increasing the photon absorption of CS itself to the maximum by adjusting parameters is our aim.

**Fig. 1:**
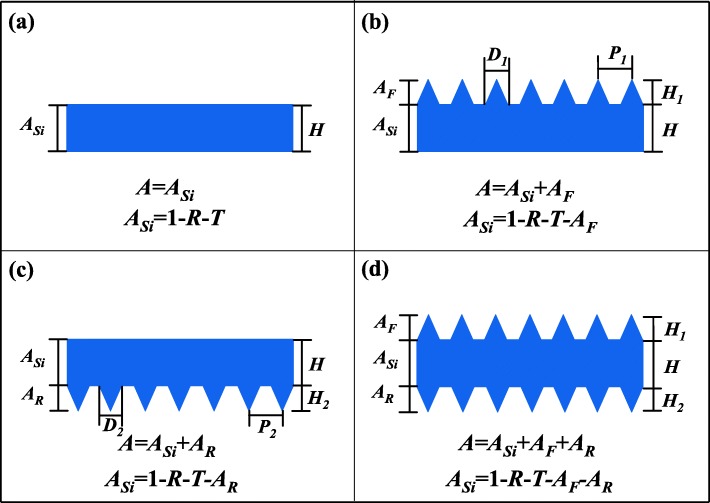
Different structures of crystalline silicon (CS) thin-film solar cell with or without pyramid gratings. **a** The bare crystal silicon (BCS). **b** The front pyramid grating (FPG). **c** The rear pyramid grating (RPG). **d** The double-sided pyramid grating (DSPG). (*A*_*F*_, *A*_*Si*_, and *A*_*R*_ represent the light absorption of the front surface gratings, the CS part, and the rear surface gratings, respectively. *H* is the thickness of the CS layer; *P*_*1*_, *D*_*1*_, *H*_*1*_ and *P*_*2*_, *D*_*2*_, *H*_*2*_ represent the period, bottom diameter, and height of silicon pyramid of the front or rear surface, respectively)

In this paper, the photon absorption distributions of the front pyramid grating (FPG), the rear pyramid grating (RPG), and the double-sided pyramid grating (DSPG) are studied. The total photon absorption *A* is further divided into three different parts as shown in Fig. 1, the photon absorption of the front surface gratings, the CS part, and the rear surface gratings and labelled as *A*_*F*_, *A*_*Si*_, and *A*_*R*_, respectively. The light reflection *R*, transmission *T*, and total absorption *A* satisfy *R* + *T* + *A* = 1. *A*_*Si*_ is not calculated in the same way for different structure models.

## Methods

**Fig. 2:**
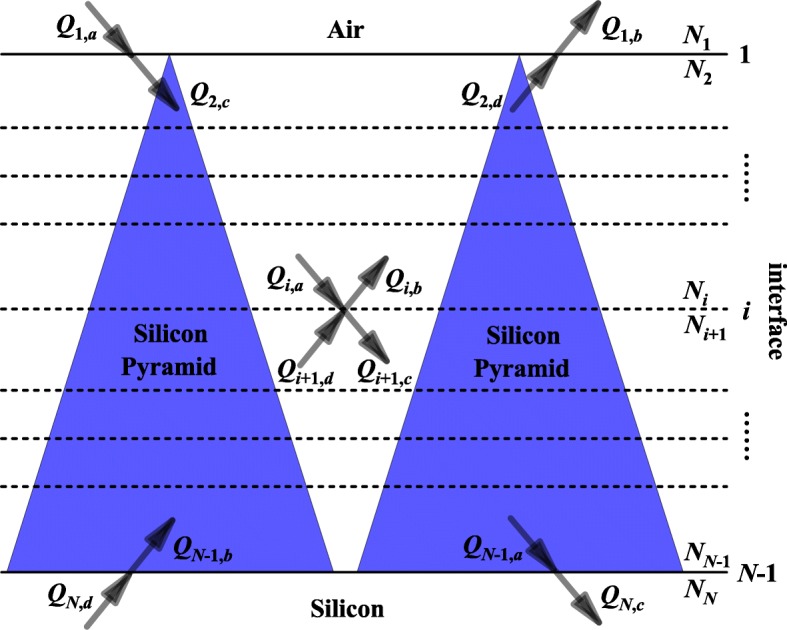
Schematic multilayer medium structure of the silicon pyramid gratings, with numbering convention of interfaces (1, …, *i*, …, *N* − 1), complex refractive index (*N*_1_, …, *N*_*i*_, …, *N*_*N*_), and electromagnetic radiation fluxes (*Q*_*i*,*a*_, *Q*_*i*,*b*_, *Q*_*i* + 1,*c*,_ *Q*_*i* + 1,*d*_, …)

In our theoretical calculations, the net radiation method and effective medium approximation are used together because of the good matching between simulation and experimental results [4, 11]. As shown in Fig. 2, a multilayer medium system of *N* layers, *N*_*i*_ is the complex refractive index of the *i*th medium and the interfaces are labeled *i* = 1, …, *N −* 1, where *i* is the total number of interfaces. Subscripts *a*, *d* and *b*, *c* represent the incoming and outgoing electromagnetic radiation, respectively. The relations between the outgoing and incoming energy fluxes (*Q*) at each interface can be expressed in terms of the reflection at the interface and the transmission passing through the medium. For every interface *i*, there are four equations,

1 $$ \left\{\begin{array}{l}{Q}_{i,a}={\tau}_i{Q}_{i,c}\\ {}{Q}_{i,b}={{r_i}_{,}}_{i+1}{Q}_{i,a}+{t}_{i+1,i}{Q}_{i+1,d}\\ {}{Q}_{i+1,c}={t}_{i,i+1}{Q}_{i,a}+{r}_{i+1,i}{Q}_{i+1,d}\\ {}{Q}_{i+1,d}={\tau}_{i+1}{Q}_{i+1,b}\end{array}\right. $$

*r*_*i*,*i* + 1_ and *t*_*i*,*i* + 1_ (*r*_*i*,*i* + 1_ + *t*_*i*,*i* + 1_ = 1) are the reflectivity and transmissivity, respectively, which are determined using Fresnel’s laws at each of the interfaces. The subscripts indicate energy fluxes transferring from layer *i* to layer *i* + 1 and vice versa. *τ*_*i*_ is the absorption attenuation rate of layer *i*, defined by

2 $$ {\tau}_i=\exp \left[-{\alpha}_i\ {d}_i/\cos \left({\varphi}_i\right)\right] $$

where *α*_*i*_ = 4π*k*_*i*_/*λ* is the absorption coefficient of layer *i* and *d*_*i*_/cos(*φ*_*i*_) is the distance travelled through the layer of thickness *d*_*i*_ with propagation angle *φ*_*i*_. *k*_*i*_ is the imaginary part of the complex refractive index *N*_*i*_ = *n*_*i*_ − *ik*_*i*_. Both the real refractive index *n*_*i*_ and the extinction coefficient *k*_*i*_ are functions of *λ*. Assuming the perpendicular incident energy flux *Q*_1,*a*_ = 1 and *Q*_*N*,*d*_ = 0, then, for each layer *i*, the energy absorption coefficient *A*_*i*_ = *Q*_*i*,*a*_ − *Q*_*i*,*c*_ + *Q*_*i*,*d*_ − *Q*_i,*b*_ can be worked out.

The effective multilayer structure of silicon pyramid is also shown in Fig. 2, and the complex refractive indices of different layers can be solved by the effective medium approximation formula,

3 $$ \frac{f_1\left({N}_{Si}^2-{N}_{Eff}^2\right)}{\left({N}_{Si}^2+2{N}_{Eff}^2\right)}+\frac{f_2\left({N}_{Air}^2-{N}_{Eff}^2\right)}{\left({N}_{Air}^2+2{N}_{Eff}^2\right)}=0 $$

where *f*_1_ and *f*_2_ are the ratio of volume filling of silicon pyramid gratings and the air, respectively, and *f*_1_ + *f*_2_ = 1. *N*_*Si*_, *N*_*Air*_, and *N*_*Eff*_ are the complex refractive indices of CS, air, and the interlayer of silicon pyramid gratings, respectively.

Combining the above formulas, the absorbed photon flux of each layer can be calculated by the following formula,

4 $$ {\varPhi}_i=\int {A}_iF\left(\lambda \right)\lambda /\left({h}_0{c}_0\right) d\lambda $$

*A*_*i*_ is the energy absorption coefficient of each layer; *F*(*λ*) is the distribution of solar radiation spectral intensity on the Earth’s surface under AM1.5 spectrum. *λ* is the wavelength of incident light, *h*_0_ and *c*_0_ are the Planck constant and speed of light in vacuum, respectively. The total number of absorbed photon can be expressed as *Φ* =  ∑ *Φ*_*i*_.

## Results and Discussion

For the different pyramid grating structures, and for comparison purposes, the related parameters are selected as follows. Firstly, the thickness of CS layer *H* = 10 μm; the height and bottom diameter of silicon pyramid are set *H*_*1*_ = *H*_*2*_ = 200 nm and *D*_*1*_ = *D*_*2*_ = 100 nm, respectively. For FPG, the ratio of period to bottom diameter is set *P*_*1*_/*D*_*1*_ = 1, and for RPG, two ratios *P*_*2*_/*D*_*2*_ = 1 and *P*_*2*_/*D*_*2*_ = 10 are considered. Finally, for DSPG, the different combinations of the above parameters are compared.

**Fig. 3:**
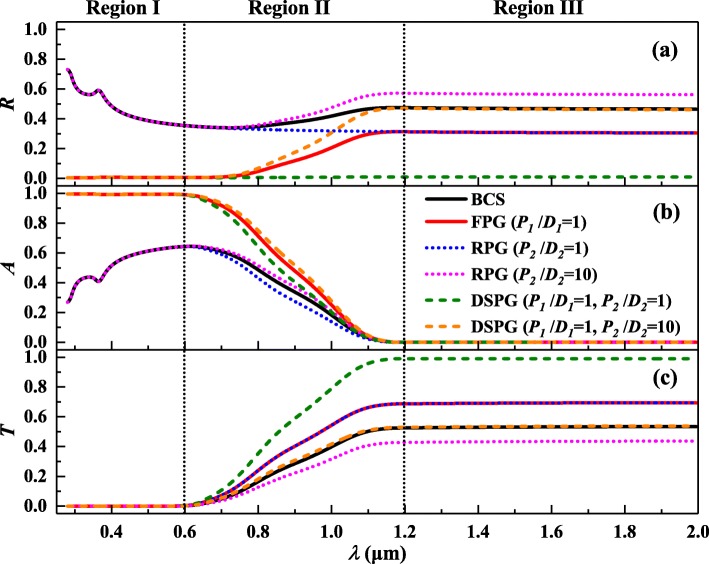
Optical properties of different silicon pyramid grating structures under the given parameters compared to the BCS of the same thickness (BCS (*H* = 10 μm), FPG (*P*_*1*_/*D*_*1*_ = 1, *H*_*1*_ = 200 nm), RPG (*P*_*2*_/*D*_*2*_ = 1 or *P*_*2*_/*D*_*2*_ = 10, *H*_*2*_ = 200 nm), DSPG (*P*_*1*_/*D*_*1*_ = 1, *P*_*2*_/*D*_*2*_ = 1 or *P*_*2*_/*D*_*2*_ = 10, *H*_*1*_ = *H*_*2*_ = 200 nm)). (**a**), (**b**), and (**c**) are the total light reflectivity, absorptivity, and transmissivity, respectively

The optical performances of different pyramid grating structures under the given parameters are shown in Fig. 3. As can be seen from Fig. 3 (a) and (b), the front surface gratings can greatly reduce the light reflection of the whole band and improve the total light absorption, especially in regions I and II. Meanwhile, in region II, the absorption of infrared light can be improved by the rear surface gratings under proper ratio parameters (*P*_*2*_/*D*_*2*_ = 10). Therefore, using them together, for DSPG, adjusting the right parameters not only can they maximize the light absorption to the Yablonovitch limit [7], but also achieve the zero light reflection of the whole band which can make the true black silicon. In addition, the rear surface pyramid gratings can increase transmission of the visible and near-infrared light seen from Fig. 3 (c), which is beneficial to be used in near-infrared photodetectors and other fields [9, 10].

**Fig. 4:**
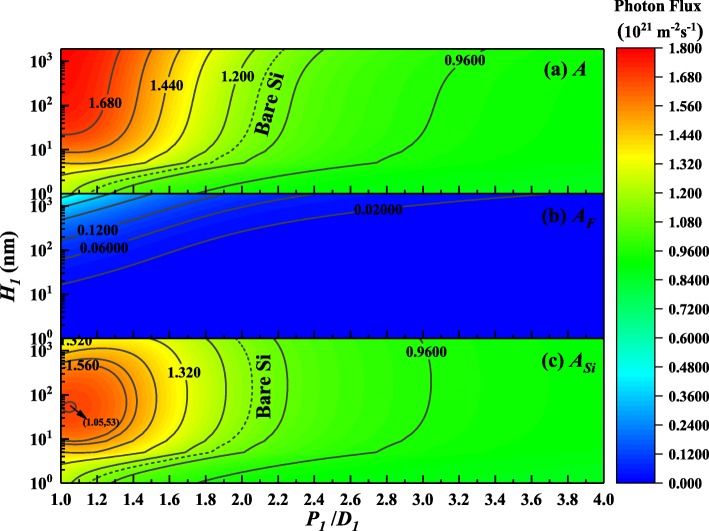
Contour maps of the photon absorption distribution in different parts for FPG structure. (**a**) The total photon absorption *A*. (**b**) The photon absorption of the front surface gratings *A*_*F*_. (**c**) The photon absorption of CS part *A*_*Si*_. (The dotted line in the illustration represents the absorption of BCS)

**Fig. 5:**
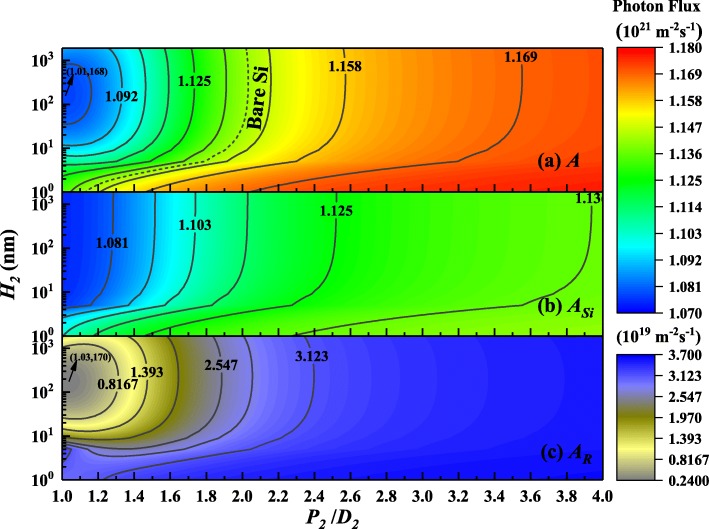
Contour maps of the photon absorption distribution in different parts for RPG structure. (**a**) The total photon absorption *A*. (**b**) The photon absorption of CS part *A*_*Si*_. (**c**) The photon absorption of the rear surface gratings *A*_*R*_. (The dotted line in the illustration represents the absorption of BCS)

For the CS solar cells, greatly enhancing the light absorption especially in the CS body is the ultimate goal. Therefore, it is necessary to study further the distribution of absorbed photons between various parts. For the FPG structure and RPG structure, three-dimensional contour maps of photon absorption in each part are shown in Fig. 4 and Fig. 5, respectively.

For the FPG structure, changing the geometric parameters of pyramid arrays, the overall photon absorption distribution compared with the photon absorption distribution of each part is shown in Fig. 4. It can be seen from Fig. 4 (a) that the total absorbed photons increase with the higher height of the pyramid, whereas the larger ratio of period to diameter is not effective for photon absorption. So, it means that the higher height and together with the smaller gap will harvest more high-frequency photons and the same seems true for the FPG absorption shown in Fig. 4 (b). However, if the height of the FPG continues to increase, it reduces the photon absorption of the CS located below as shown in Fig. 4 (c). Obviously, there is an optimal parameter configuration where *P*_*1*_/*D*_*1*_ = 1.05, *H*_*1*_ = 53 nm. Furthermore, if it is assumed that the photons absorbed by the silicon pyramid are not involved in the conversion of electron-hole pairs in the CS, based on these calculations, the suitable ranges of the FPG geometric parameters are also obtained and compared with the bare silicon shown in Fig. 4 (c). In short, the higher the height of FPG, the lower the reflectivity, but this does not mean there is more effective light absorption.

In the same way, for the RPG structure, the photon absorption distributions of the whole and each part are shown in Fig. 5. For the total absorption shown in Fig. 5 (a), compared with the FPG structure, there shows a significant difference in that photon absorption is enhanced with the larger ratio of period to bottom diameter and the lower pyramid height. This means that, on one hand, the larger ratio of *P*_*2*_/*D*_*2*_ and smaller *H*_*2*_ reduce the low-frequency photon transmission and the photons turn back, thereby increasing the reflection. But, on the other hand, photons are promoted to be absorbed in the process. Obviously, the parameter configuration which results in the least absorption is *P*_*2*_/*D*_*2*_ = 1.01, *H*_*2*_ = 168 nm, and the suitable ranges of the RPG geometric parameters are also obtained compared with the bare silicon shown in Fig. 5 (a). However, on the CS part shown in Fig. 5 (b), there is no obvious improvement in the effective light absorption because a large number of photons are reflected. Figure 5 (c) shows that the photons absorbed by the rear surface grating are two orders of magnitude lower than those absorbed by CS, and there is a similar trend which looks like that of the total absorption shown in Fig. 5 (a). Here also, the parameters configuration are *P*_*2*_/*D*_*2*_ = 1.03 and *H*_*2*_ = 170 nm and almost the same as above.

**Fig. 6:**
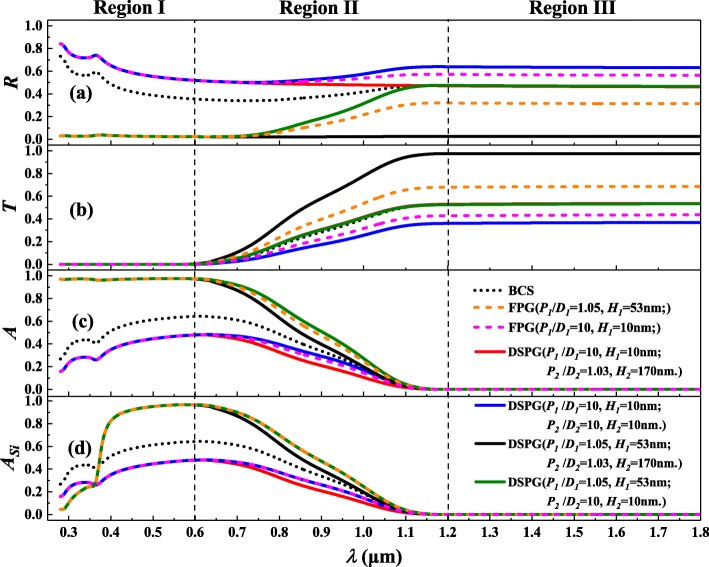
Optical properties of four sets of different parameters for the DSPG (*P*_*1*_/*D*_*1*_ = 10, *H*_*1*_ = 10 nm and *P*_*2*_/*D*_*2*_ = 1.03, *H*_*2*_ = 170 nm or *P*_*2*_/*D*_*2*_ = 10, *H*_*2*_ = 10 nm; *P*_*1*_/*D*_*1*_ = 1.05, *H*_*1*_ = 53 nm and *P*_*2*_/*D*_*2*_ = 1.03, *H*_*2*_ = 170 nm or *P*_*2*_/*D*_*2*_ = 10, *H*_*2*_ = 10 nm) compared to the BCS (*H* = 10 μm) and FPG (*P*_*1*_/*D*_*1*_ = 1.05, *H*_*1*_ = 53 nm and *P*_*1*_/*D*_*1*_ = 10, *H*_*1*_ = 10 nm). (**a**), (**b**), (**c**), and (**d**) are the total light reflectivity, transmissivity, absorptivity, and the absorptivity of CS part, respectively

As seen from the absorption distribution of the FPG and the RPG, the former obviously plays an important role in improving the photon absorption shown in Fig. 4 (c), while the latter implies that photon absorption in the CS part is weakened because of the existence of the rear surface gratings shown in Fig. 5 (b). Combining the findings above, the optical properties of the four sets of different parameters which are representative of the DSPG are studied and shown in Fig. 6.

Due to the weak transmission ability of high-frequency photons shown in Fig. 6 (b), if the ratio of period to bottom diameter is not appropriate (*P*_*1*_/*D*_*1*_ = 10 and *H*_*1*_ = 10 nm), not only does it not reduce the reflectivity, but it also causes the reflection to increase and the absorption to decrease as shown in Fig. 6. Only suitable parameters (*P*_*1*_/*D*_*1*_ = 1.05 and *H*_*1*_ = 53 nm) can achieve significant enhancement of light absorption. For the CS, because of its own inability to absorb the lower frequency photons as shown in the region III, the modulation of the front and rear surface gratings only affects the distribution of light between the reflection and transmission. It becomes obvious that the rear gratings are playing a major role in region II and region III, and with the suitable match of the front surface gratings parameters (*P*_*1*_/*D*_*1*_ = 1.05, *H*_*1*_ = 53 nm, and *P*_*2*_/*D*_*2*_ = 1.03, *H*_*2*_ = 170 nm), nearly zero reflection of full wave band can be realized. Compared with the FPG of the same parameters, for the total absorption shown in Fig. 6 (c), in region II, the presence of the rear surface gratings with appropriate parameters can actually improve the infrared light absorption (*P*_*2*_/*D*_*2*_ = 10, *H*_*2*_ = 10 nm), which confirms previous conclusions that the mismatched double grating design can enable significant improvements in device performance [10]. However, for the absorption of the CS part shown in Fig. 6 (d), using the design of rear surface gratings has little effect in improving light absorption of CS. Therefore, in this sense, although the RPG can reflect light and redirect it back toward the photoactive regions of the solar cell [12], it does provide no added benefit for the effective light absorption. Some novel designs to tune absorption spectrum for an optimized integration need to be developed [1, 13].

## Conclusions

The design of double-sided pyramid grating structure is adopted to promote the overall light absorption of the silicon solar cell, and it can also realize the zero reflection by adjusting the parameters. However, for the effective light absorption of CS part, it does not increase with the enhancement of the overall light absorption. For the front surface pyramid gratings, the suggested ratio of *P*_*1*_/*D*_*1*_ is less than 1.4 and *H*_*1*_ is between 10 and 600 nm, and for the rear surface pyramid gratings, there is little effect on the effective light absorption enhancement, so no rear gratings are necessary. Therefore, the innovation and optimized design of the front surface texture is a big trend for further improvement of solar cell efficiency.

## Abbreviations

CS: Crystalline silicon
DSPG: Double-sided pyramid grating
FPG: Front pyramid grating
RPG: Rear pyramid grating
